# *In Vitro* Resistance Selections for *Plasmodium falciparum* Dihydroorotate Dehydrogenase Inhibitors Give Mutants with Multiple Point Mutations in the Drug-binding Site and Altered Growth* [Fn FN2]

**DOI:** 10.1074/jbc.M114.558353

**Published:** 2014-04-29

**Authors:** Leila S. Ross, Francisco Javier Gamo, Maria José Lafuente-Monasterio, Onkar M. P. Singh, Paul Rowland, Roger C. Wiegand, Dyann F. Wirth

**Affiliations:** From the ‡ Department of Immunology and Infectious Diseases, Harvard School of Public Health, Boston, Massachusetts 02115,; the § Tres Cantos Medicines Development Campus, GlaxoSmithKline, 28760 Tres Cantos, Madrid, Spain,; the ¶ Computational and Structural Chemistry, GlaxoSmithKline, Stevenage, Hertfordshire SG1 2NY, United Kingdom, and; the ‖ Infectious Disease Initiative, Broad Institute, Cambridge, Massachusetts 02142

**Keywords:** Drug Resistance, Evolution, Infectious Disease, Malaria, Nucleotide, Pyrimidine

## Abstract

Malaria is a preventable and treatable disease; yet half of the world's population lives at risk of infection, and an estimated 660,000 people die of malaria-related causes every year. Rising drug resistance threatens to make malaria untreatable, necessitating both the discovery of new antimalarial agents and the development of strategies to identify and suppress the emergence and spread of drug resistance. We focused on in-development dihydroorotate dehydrogenase (DHODH) inhibitors. Characterizing resistance pathways for antimalarial agents not yet in clinical use will increase our understanding of the potential for resistance. We identified resistance mechanisms of *Plasmodium falciparum* (Pf) DHODH inhibitors via *in vitro* resistance selections. We found 11 point mutations in the PfDHODH target. Target gene amplification and unknown mechanisms also contributed to resistance, albeit to a lesser extent. These mutant parasites were often hypersensitive to other PfDHODH inhibitors, which immediately suggested a novel combination therapy approach to preventing resistance. Indeed, a combination of wild-type and mutant-type selective inhibitors led to resistance far less often than either drug alone. The effects of point mutations in PfDHODH were corroborated with purified recombinant wild-type and mutant-type PfDHODH proteins, which showed the same trends in drug response as the cognate cell lines. Comparative growth assays demonstrated that two mutant parasites grew less robustly than their wild-type parent, and the purified protein of those mutants showed a decrease in catalytic efficiency, thereby suggesting a reason for the diminished growth rate. Co-crystallography of PfDHODH with three inhibitors suggested that hydrophobic interactions are important for drug binding and selectivity.

## Teaser

**Background:** Inhibiting PfDHODH kills malaria parasites, but the potential for drug resistance is unknown.

**Results:** Selections gave several categories of resistance mutations. Several mutants were hypersensitive to other drugs.

**Conclusion:** Resistance to PfDHODH inhibitors is largely though mutations in or amplification of the target gene, PfDHODH.

**Significance:** Resistance to PfDHODH inhibitors is possible but often increases sensitivity to other compounds.

## Introduction

Malaria is a mosquito-borne disease caused by infection with *Plasmodium* parasites. In 2010, there were an estimated 219 million cases, resulting in 660,000 malaria-related deaths (1). Children under the age of 5 bear the heaviest burden from malaria morbidity and mortality.

Resistance has compromised nearly all therapies used for malaria (2), including reduced efficacy of the current front-line artemisinin combination therapies (3). The control and eradication of malaria require a steady supply of affordable and effective antimalarial drugs that are safe for entire populations, including pregnant women, infants, and people with hemoglobinopathies common in malaria-endemic regions such as glucose-6-phosphate dehydrogenase deficiency. Drug resistance complicates this already lofty goal. Given that an infected person may harbor 10^10^–10^13^ parasites in his or her bloodstream and that there are an estimated 200–500 million cases of malaria per year, the potential for resistance is enormous (4). Additionally, drug resistance can spread locally within a single transmission season and globally in a few years (2). New therapies must take potential resistance into account or risk a quick obsolescence.

Methods to limit resistance have largely relied on combination therapy, where the driving concept is that it is difficult to become resistant to two compounds in the same time frame. Evolutionary fitness constraints limit the diversity of resistance pathways in a population. For example, resistance to pyrimethamine in *Plasmodium falciparum* is best accomplished with a set of four mutations in the dihydrofolate reductase gene. Although there are 24 possible orders of mutation, three pathways account for 90% of observed resistance, and all veer to the same outcome of four specific mutated residues (5). Similarly, a limited number of paths to resistance were followed with high probability for bacterial β-lactamase inhibitors, indicating that this is a widespread phenomenon that applies to both prokaryotes and eukaryotes (5). Over time, compensatory mutations can restore fitness (6); this expands the number of possible resistance pathways. Thus, acting early to prevent the initial emergence of resistance may restrict parasite options to those few heavily favored, highly fit pathways. These pathways can be predicted through *in vitro* selection experiments (7) and preemptively blocked through the development of mutant-selective inhibitors.

Identifying and combining antimalarial compounds that selectively target the bulk of the wild-type population and the small, emerging resistant population are novel approaches to antimalarial combination therapy. We tested this idea, coined “targeting resistance,” with inhibitors of pyrimidine biosynthesis.

Pyrimidines, thiamine (vitamin B_1_) and the nucleobases thymine, cytosine, and uracil, are ubiquitous and essential in cells. There are two ways to obtain pyrimidines: salvage and *de novo* synthesis. Malaria parasites lack pyrimidine salvage pathways and are completely reliant upon *de novo* synthesis (8). The enzyme dihydroorotate dehydrogenase (DHODH)^2^ catalyzes the rate-limiting step of pyrimidine biosynthesis. Crystal structures showed significant differences between the human and *P. falciparum* DHODH enzymes (9, 10), and several groups have developed inhibitors specific for the human or malarial enzymes (11, 12).

We performed *in vitro* resistance selections with PfDHODH inhibitors against wild-type parasites. Characterization of the resulting resistant lines revealed six point mutations in the PfDHODH target as follows: E182D, F188I, F188L, F227I, I263F, and L531F. Target gene amplification also gave resistance, but this occurred less often than mutation and had a smaller effect.

Testing resistant mutant parasites against a set of PfDHODH inhibitors revealed an interesting pattern; although cross-resistance was observed for related compounds, different structural classes of PfDHODH inhibitors were often unaffected or even more potent against the mutant parasites than the parental wild type, a phenomenon referred to as negative cross-resistance. Negative cross-resistance implies that the two compounds have mutually incompatible resistance mechanisms, *i.e.* an organism cannot be resistant to both at the same time.

We carried out additional resistance selections with mutant parasites and these negative cross-resistant, mutant-selective inhibitors. These sequential selections gave rise to three more point mutations in the target gene as follows. In the F227I mutant line, separate selections led to the additional mutations L172F and L527I, and in the E182D mutant line, a second mutation in codon 182 restored the wild-type protein sequence (E182D and D182E), albeit with an alternate codon. Comparative growth assays demonstrated that the E182D mutant and the E182D and D182E wild-type revertant both grew poorly when compared with the wild-type 3D7 parent, but they had similar growth rates to each other. Simultaneously selecting parasites with a combination of wild-type and mutant-type selective PfDHODH inhibitors, a test of targeting resistance, maintained a wild-type population and prevented the emergence of resistance far more often than selection with either drug alone. In contrast, simultaneously selecting parasites with compounds that did not exhibit mutually incompatible resistance did not protect from the development of resistance.

Many of the resistance mutations in *pfdhodh* are located near the 5′ end and are thus difficult to genetically alter via traditional allelic exchange strategies. In lieu of genetic evidence, we pursued biochemical approaches. Recombinant PfDHODH protein recapitulated the drug responses seen in mutant parasites, suggesting that point mutations in *pfdhodh* are the main factor in drug resistance. PfDHODH E182D protein had lower catalytic efficiency than the wild-type protein, which may provide an explanation for the mutant parasite's altered growth. Crystallography of wild-type PfDHODH protein with three inhibitors implied that selectivity is largely due to hydrophobic interactions rather than hydrogen bonding or steric clashes.

## EXPERIMENTAL PROCEDURES

### 

#### 

##### Parasite Culture

The erythrocytic stages of *P. falciparum* were grown at 37 °C in solutions of 4% O^+^ hematocrit in RPMI 1640 medium supplemented with 28 mm NaHCO_3_, 25 mm HEPES, and 5% albumax II (w/v). Human blood was supplied from Research Blood Components or Interstate Blood Bank. Cultures were grown in a gas mixture of 5% O_2_, 5% CO_2_, and 90% N_2_. Cultures were maintained with media changes every other day and were subcultured to maintain parasitemia below 4%. At least 25% of the hematocrit was replaced weekly. Parasite growth was synchronized by treatment with sorbitol (13). Frozen stocks were prepared using a solution composed of 28% glycerol, 3% sorbitol, and 0.65% sodium chloride. The Dd2 parasite used was a clone derived from MR4 line MRA-156.

##### Resistance Selection

Approximately 1 × 10^9^ clonal ring stage parasites were divided among four independent 25- ml culture flasks with 4% hematocrit and 4% parasitemia each. These parasites were treated with 10 times the IC_50_ of a compound for 2 days. For all simultaneous selections, 10 times the IC_50_ of each compound was used, and synergy was assessed via isobologram. Media and drug were replenished on both days. Compound pressure was then removed, and the cultures were fed on alternate days with compound-free complete RPMI 1640 media. 25% of the hematocrit was replaced weekly if no parasites were visible by thin blood smear. Compound exposure was repeated after parasites reappeared in culture. These steps were executed for 30–100 days. A selection was discarded if no parasites grew after 80 days of recovery. Selected parasites were cloned by limiting dilution in a 96-well plate with an inoculum of 0.2 infected red blood cells per 100-μl well. Parasite clones were detected after 2.5 weeks of growth by microscopy.

##### Genomic DNA Analysis

Genomic DNA was extracted from parasites using the QIAamp blood kit (Qiagen) for sequence analysis of the *pfdhodh* gene (PlasmoDB code Pf3D7_0603300; GenBank^TM^ accession number AB070244.1; and NCBI reference sequence XM_960930.1). A 2.2-kb fragment encompassing the complete *pfdhodh* ORF was PCR-amplified from drug-resistant clones and parental lines (primer sequences below). PCR-amplified fragments were fully sequenced using *pfdhodh*-specific primers as follows: PfDHODH forward 5′-GATCCCTAGGATGATCTCTAAATTGAAACCTCAATTTATG-3′, PfDHODH reverse 5′-GATACTCGAGTTAACTTTTGCTATGCTTTCGGCCAATG-3′, and PfDHODH internal 5′-CATTATTTGGATTATATGGTTTTTTTGAATCTTATAATCCTG-3′.

The *pfdhodh* gene was amplified with Pfu Ultra II Fusion DNA polymerase (Agilent) with the following conditions: step 1, 95 °C for 120 s; step 2, 95 °C for 30 s; step 3, 55 °C for 30 s, and step 4, 60 °C for 140 s, go to step two and repeat 30 times. PCR amplicons were purified with a PCR clean-up kit (Qiagen) and sequenced. Cytochrome *b* (PlasmoDB code mal_mito_3) and cytochrome *c* oxidase (PlasmoDB code mal_mito_1) were amplified and sequenced with the following primers: Mal_mito_1 forward 5′-GTAATTTATCAAATATAAAAGCACATCTAGTTTC-3′ and Mal_mito_1 reverse 5′-CTGAATAGAATAAGAACTCTATAAATAACCAG-3′; Mal_mito_3 forward 5′-GCTCATGAACTTTTACTCTATTAATTTAGTTAAAGC-3′ and Mal_mito_3 reverse 5′-GATCTTATATGTTTGCTTGGGAGCTG-3′.

##### Whole Genome Sequencing (for PfDHODH E182D, D182E, and F227I Lines)

Genomic DNA was sheared and made into a 200-bp fragment Illumina sequencing library and sequenced with paired-end reads on an Illumina GAIIx machine at the Broad Institute (Cambridge, MA).

##### Whole Genome Sequencing (for Dd2 Clone AL and Several Parasites with Multiple Copies of pfdhodh)

One microgram of genomic DNA in 50 μl of water was sheared with a Covaris sonicator. 300 ng of sheared DNA in 15 μl of water was used to create libraries in an Apollo324 machine (IntegenX) using adaptors from BioO NEXTFlex. Library quality was assessed with a bioanalyzer DNA chip and quantitative PCR (ABI Prism Illumina kit, KAPA Biosystems). These Illumina sequencing libraries were prepared at the Harvard Bauer Core facility and sequenced with paired-end 2 × 250-bp MiSeq at the Broad Institute.

##### Whole Genome Sequencing Analysis

Sequenced reads were aligned against the *P. falciparum* 3D7 reference from PlasmoDB (version 7.1*) (14) using BWA version 0.5.7 (15). Duplicate reads were marked using Picard MarkDuplicates. Consensus bases were called using the Genome Analysis Toolkit (GATK) Unified Genotyper (version 1.0.5974) (16) and the SAMtools (version 0.1.16) (17) mpileup command. Only bases that were called as homozygous for the reference or the alternate allele with a genotype quality of at least 30 was considered.

##### In Vitro Drug Sensitivity and IC_50_ Determinations

Drug susceptibility was measured using the SYBR Green method (18), and compounds were printed onto 384-well plates using a D300 digital dispenser (Hewlett-Packard). Compounds were stored dry at −20 °C for long term storage. Compounds were dissolved in DMSO for use, and these stocks were arrayed in single-use aliquots and stored at −80 °C to reduce the risk of compound degradation.

Twelve point curves based on 2-fold dilutions of the test compound were carried out in triplicate and replicated with three different parasite cultures. IC_50_ values were calculated using the log(inhibitor) *versus* response-variable slope equation in GraphPad Prism version 5.0d (Equation 1),

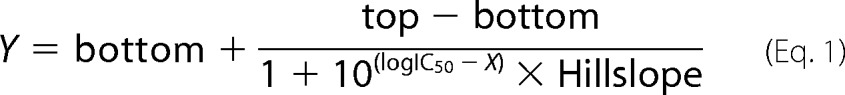
 where *X* = concentration of compound, and *Y* = normalized percent viability.

##### Synthesis of DSM74

The compound DSM74 was prepared following the literature procedure (19) and was recrystallized from ethanol. ^1^H NMR spectra matched reported results, and HPLC analysis indicated >95% purity.

##### Copy Number Variation Quantitative PCR

PfDHODH copy number was assessed using the quantitative PCR primers and controls described in Guler *et al.* (20). Power SYBR Green master mix with ROX (Applied Biosystems) was used on a 7900 HT quantitative PCR machine (Applied Biosystems). Relative copy number was determined for 1 ng of genomic DNA using the ΔΔ*C_T_* method (21). The relative copy number of the multidrug resistance transporter *pfmdr1* was determined for 0.1 ng of genomic DNA using the following gene-specific primers, and it was normalized using the same seryl tRNA synthetase and 18 S ribosomal rDNA control primers used for the above *pfdhodh* quantitative PCRs as follows: PfMDR1 forward 5′-TGCATCTATAAAAC GATCAGACAAA-3′ and PfMDR1 reverse 5′-TCGTGTGTTCCATGTG ACTGT-3′,

##### Plasmid Construction and Mutagenesis

A synthetic codon-optimized gene encoding residues 159–565 of PfDHODH (9) (GenBank^TM^ accession number AY685129) was cloned into a pET28b plasmid with an amino-terminal His_6_ tag followed by a recombinant tobacco etch virus cleavage site as described by Deng *et al.* (22), resulting in pET28b-His_6_-rTEV-PfDHODH del(1–158). The listed mutations were created with the following primers and cloned into the tagged pET28b construct described above. The N61S/G254A double mutant was not made because residue 61 is not part of the heterologous expression plasmid due to solubility issues, and there is thus no complete model for this mutant protein as follows: L172F forward 5′-CCTGTACGATATTTTCTTCAAATTTTGTTTGAAGTA-3′ and L172F reverse 5′-GTACTTCAAACAAAATTTGAAGAAAATATCGTACA-3′; E182D forward 5′-GTACATCGATGGTGACATTTGCCATGACCTG-3′ and E182D reverse 5′-CAGGTCATGGCAAATGTCACCATCGATGTAC-3′; F188I forward 5′-AGCCATGACCTGATTTTGCTGCTTGG-3′ and F188I reverse 5′-CAAGCAGCAAAATCAGGTCATGGCT-3′; and F188L forward 5′-ATTTGCCATGACCTGCTGTTGCTGCTTGG-3′ and F188L reverse 5′-CCAAGCAGCAACAGCAGGTCATGGCAAAT-3′; F227I forward 5′-GTTGCAGCTGGAATCGATAAAAACGGTG-3′ and F227I reverse 5′-CACCGTTTTTATCGATTCCAGCTGCAAC-3′; I263F forward 5′-GAAACCGCGGTTCTTTCGTGACGTC-3′ and I263F reverse 5′-GACGTCACGAAAGAACCGCGGTTTC-3′; L527I forward 5′-CTTCCGTGTGTCAGATCTATTCGTGCTT-3′ and L527I reverse 5′-AAGCACGAATAGATCTGACACACGGAAG-3′; and L531F forward 5′-GCTCTATTCGTGCTTCGTTTTCAACGGTATG-3′ and L531F reverse 5′-CATACCGTTGAAAACGAAGCACGAATAGAGC-3′.

##### PfDHODH Protein Expression and Purification

BL21(DE3)Star *Escherichia coli* (Invitrogen) were transformed with pET28b-PfDHODH expression constructs. Transformed cells were grown in Terrific Broth with 100 μg/ml kanamycin at 37 °C and 180 rpm and then dropped to 20 °C and 150 rpm for induction. Cultures were induced with 200 μm isopropyl d-thiogalactoside at *A*_600_ = 0.6–0.8. Cultures were grown 12–16 h post-induction, then pelleted by centrifugation at 10,000 × *g*, and frozen at −80 °C for later use. For purification, thawed pellets were resuspended in buffer A (100 mm HEPES, pH 8.0, 150 mm NaCl, 10% glycerol, 0.05% Thesit). The cells were lysed by sonication on ice (Branson digital sonifier 450, 5 min at 40% amplitude with a pattern of 5 s on and 10 s off). The cell lysate was clarified by centrifugation at 40,000 × *g* for 45 min at 4 °C and then filtered through a 0.45-μm filter. The clarified and filtered lysate was applied to a HisPrep FF 16/10 column (GE Healthcare) pre-equilibrated with buffer A on an AKTA Purifier FPLC. The column was washed with 30 column volumes of buffer A and then eluted with stepwise increments of buffer B (100 mm HEPES, pH 8.0, 150 mm NaCl, 10 glycerol, 0.05% Thesit, 400 mm imidazole). The eluted protein was concentrated by centrifugation in concentrator spin columns (Pierce) and then injected into a HiLoad 16/60 Superdex 200 size exclusion column pre-equilibrated with size exclusion buffer (10 mm HEPES, pH 7.8, 100 mm NaCl, 5% glycerol, 1 mm *N,N*-dimethyldodecylamine *N*-oxide, 10 mm DTT added fresh). Fractions containing PfDHODH were pooled and concentrated. Protein concentration was assessed by Bradford assay (Pierce). Protein aliquots were flash-frozen in liquid nitrogen and stored at −80 °C.

##### PfDHODH Biochemical Assays

Substrate-dependent inhibition of recombinant PfDHODH protein was assessed in an *in vitro* assay in 384-well clear plates (Corning 3702) as described by Malmquist *et al.* (23). A 16-point dilution series of inhibitor concentrations was assayed against 2–10 nm protein with 500 μm l-dihydroorotate substrate (excess), 18 mm dodecylubiquinone electron acceptor (∼*K_m_*), and 100 μm 2,6-dichloroindophenol indicator dye in assay buffer (100 mm HEPES, pH 8.0, 150 mm NaCl, 5% glycerol, 0.5% Triton X-100). Assays were incubated at 25 °C for 20 min and then assessed via *A*_600_. Atmospheric oxygen, which can act as an electron acceptor, was depleted through the addition of 0.1 mg/ml glucose oxidase, 0.02 mg/ml catalase, and 50 mm glucose, followed by incubation at 25 °C for 5 min prior to assay assembly. Data were normalized to 1% DMSO and excess inhibitor (500 nm Genz-669178 unless another compound or concentration was appropriate). Steady-state kinetic parameters were determined as described by Malmquist *et al.* (23) by direct assessment of the production of orotic acid (ϵ_296_ = 4.3 mm^−1^ cm^−1^) in UV-transparent 384-well plates (Corning 3672). For enzyme oxidase activity, 100 nm enzyme was assayed against 5–500 μm l-DHO. For oxidant affinity, 5–50 nm enzyme was assayed against a range of CoQ_d_ (1–150 μm) with excess substrate (500 μm l-DHO). Data were fitted to the Michaelis-Menten equation in GraphPad Prism 5.0d (Equation 2),

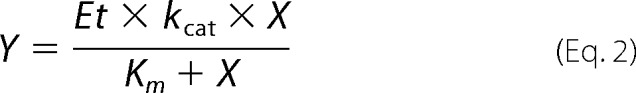
 where *Y* = enzyme velocity; *E_t_* = total enzyme concentration, and *X* = substrate concentration.

##### Comparative Fitness Assays and Allelic Discrimination Quantitative PCR

Parasite lines were synchronized with sorbitol for two life cycles (13). 2-ml cultures were seeded with a mixture of two parasite lines with 1% total parasitemia in 4% hematocrit. Cultures were grown at 37 °C with 40 rpm shaking. 90% of the cultures was taken every generation to isolate genomic DNA. The genomic DNA was analyzed by allelic discrimination quantitative PCR with the primers and probes listed in Table 1; the polymorphism site is underlined (Invitrogen). Two biological replicates, each done in triplicate, were carried out for six generations. Allelic discrimination assays were performed with TaqMan Universal PCR Master Mix with no AmpErase UNG (Applied Biosystems) on a 7900 HT real time PCR machine (Applied Biosystems). PCR cycling conditions were 95 °C for 10 min, followed by 40 cycles of 95 °C for 15 s and 56 °C for 60 s.

**TABLE 1:**
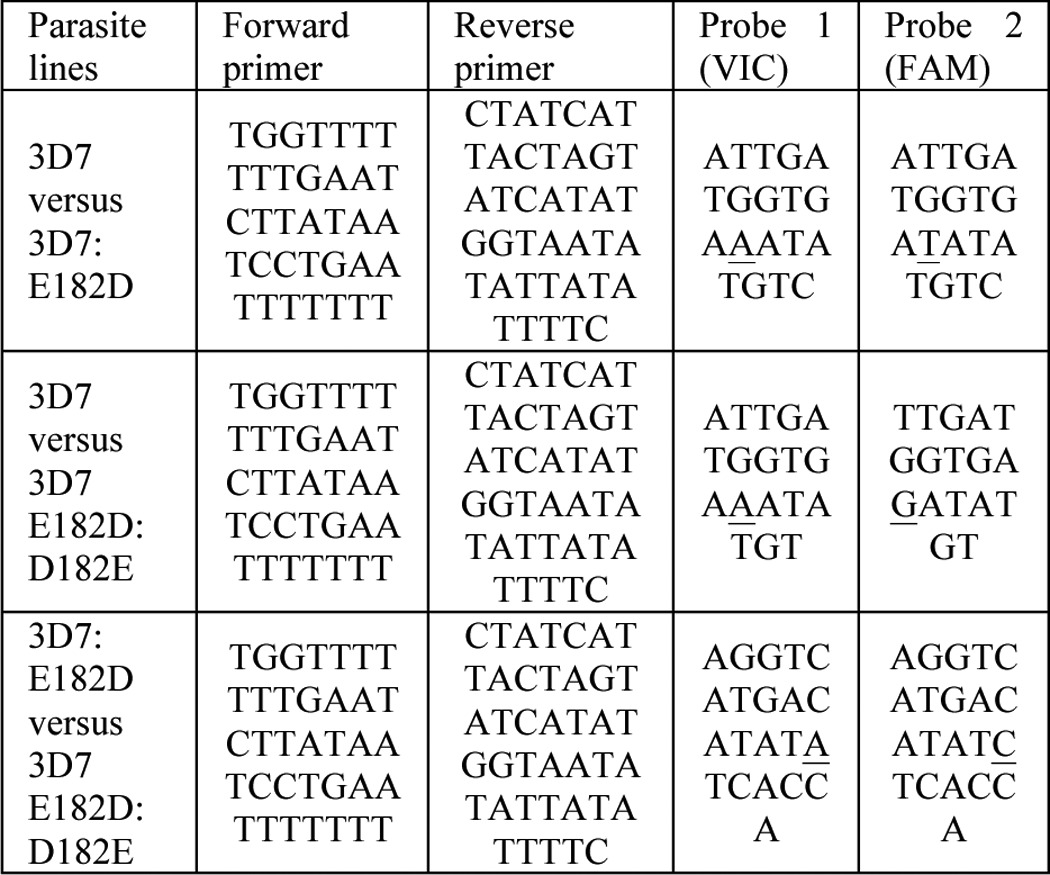
**Primers and probes for allelic discrimination assay**

##### PfDHODH Crystallography

PfDHODH protein (amino acids 158–569, Δ384–413) was produced as described (22) by GlaxoSmithKline (Stevenage, UK). Residues 1–157 were deleted as they contain a membrane anchor that is not amenable to soluble expression. Residues 384–413 were deleted as they form a disordered loop that greatly reduces the likelihood of crystal formation. Protein-compound complexes were formed by mixing 50 μl of protein (in size exclusion buffer at 10 mg/ml) with 1 μl of 500 mm *N,N*-dimethyldodecylamine *N*-oxide, 0.5 μl of 200 mm l-dihydroorotate, and 0.5 μl of 200 mm compound. Complexes were allowed to form at room temperature for 5 min before being distributed into hanging drop or paraffin oil immersion microbatch trays by hand. 1 μl of protein-compound complex was mixed with 1 μl of precipitant in each drop. All trays were incubated at 20 °C. Crystals were harvested from the following conditions: PfDHODH + Genz-669178, 0.2 m LiCl + 24% PEG 3350; PfDHODH + IDI-6253, 0.2 m LiCl + 20% PEG 3350; and PfDHODH + IDI-6273, 0.1 m MES, pH 6.5, + 20% PEG 3350. X-ray data were collected at ESRF (ID29) and Diamond (I03). Molecular models of the data are presented with CCP4mg (24).

##### Statistical Analysis

Data are shown as mean ± S.D. and were analyzed by one-way analysis of variance with Dunnett's multiple comparison post hoc test via GraphPad Prism 5.0d software. Differences were considered significant for *p* < 0.05.

## RESULTS

### 

#### 

##### Strategies for in Vitro Resistance Selections

**SCHEME 1.**
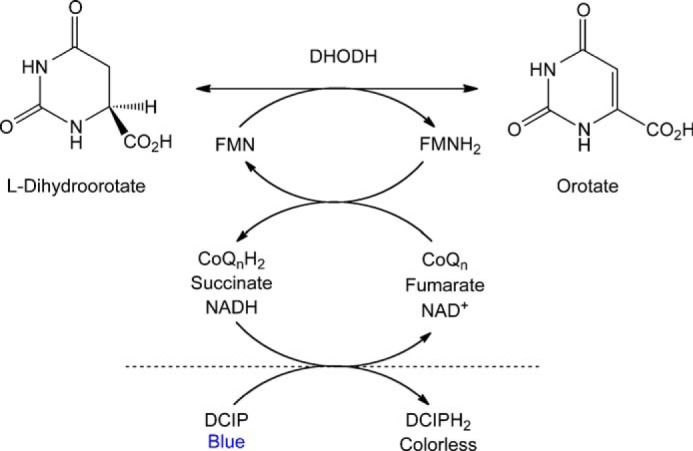
**Reactions catalyzed by DHODH.** DHODH catalyzes two reversible half-reactions. In the first, l-DHO is oxidized by the FMN cofactor. In the second, the reduced FMN cofactor is re-oxidized by a variety of electron acceptors such as fumarate or NAD^+^ in cytosolic class 1 enzymes or CoQ*_n_* in mitochondrial class 2 enzymes. Humans and *Plasmodium* parasites both have class 2 enzymes. The reduced electron acceptor may be re-oxidized by coupling to dichloroindophenol (*DCIP*), an indicator dye, to indirectly measure enzyme activity. Alternatively, the production of orotate may be directly measured.

**SCHEME 2.**
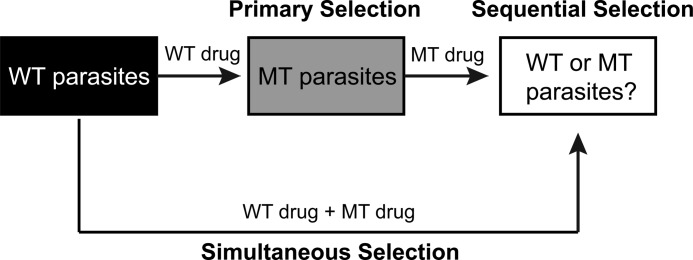
**Three types of resistance selections.** A primary selection treats wild-type parasites with a wild-type drug. If resistant parasites arise, these mutant parasites can be sequentially treated with a mutant-selective drug. A simultaneous selection treats wild-type parasites with both wild-type and mutant-type-selective drugs at the same time. *MT-type*, mutant.

PfDHODH catalyzes the rate-limiting step of pyrimidine biosynthesis, a set of reversible redox half-reactions (Scheme 1). Parallel efforts by multiple groups have led to the development of several *Plasmodium*-selective DHODH inhibitors (Table 2), including mutant-selective inhibitors that target parasites resistant to traditional wild-type-selective inhibitors (25). We sought to comprehensively sample potential resistance pathways to PfDHODH inhibitors, and we did so through 59 independent *in vitro* drug resistance selections, representing 16 sets of parasite-drug pairs (Scheme 2).

**TABLE 2:**
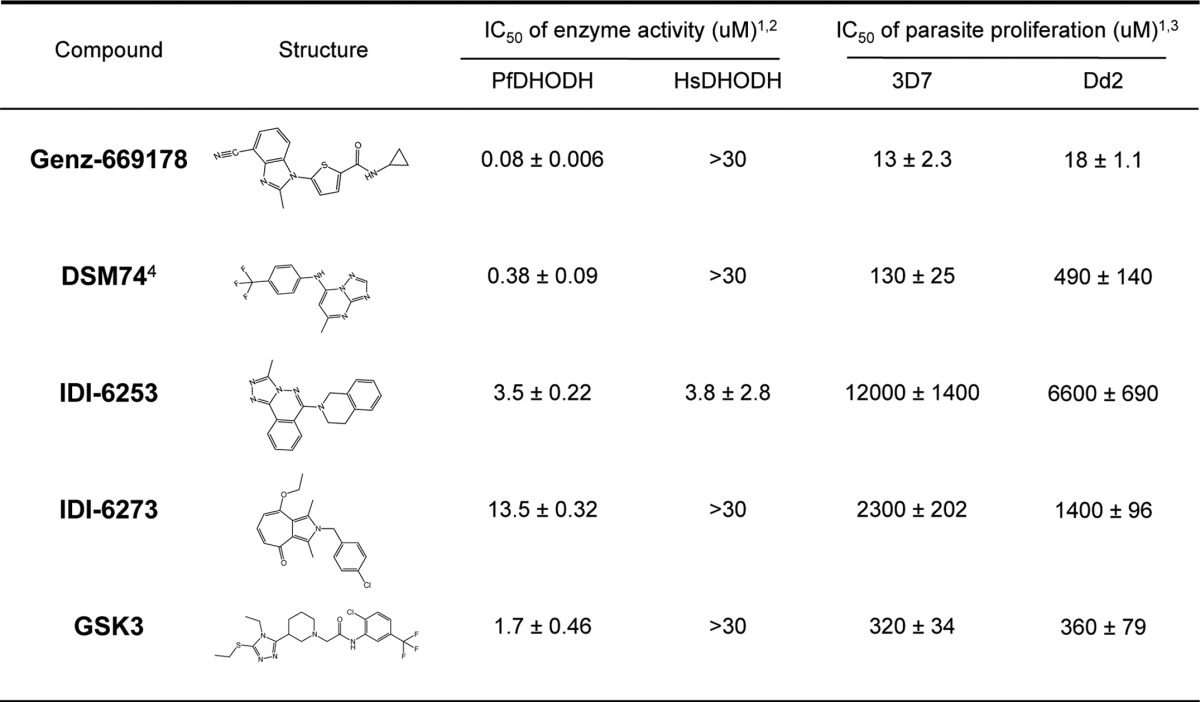
**Chemical entities found to inhibit parasites with wild-type or mutant PfDHODH**

^1^ Errors represent the standard error of the fit for at least three determinations using 12 different drug concentrations.

^2^ IC_50_ values were determined using a continuous assay where the reagent concentrations were 200 μml-DHO, 18 μm CoQ_d_, 100 μm dichloroindophenol (DCIP), and 2–10 nM DHODH.

^3^ Antimalarial efficacy was calculated from dose-effect curves based on the SYBR Green method (18).

^4^ DSM74 was developed by Professor Margaret Phillips at the University of Texas at Southwestern (11).

We chose to use a large number of parasites, approximately one billion, for each resistance selection. All selections were started from early freezes of clonal parasite populations to reduce pre-existing variation. An analysis of multiple selections primarily linked the evolution of resistance to the number of mitotic divisions (26). The number of mitotic divisions reflects both the number of parasites as well as the number of cell divisions, *i.e.* the amount of time spent in culture. We used high dose intermittent drug pulses and allowed parasite populations to fully recover between pulses in an effort to have the maximum population size going through the maximum number of bottleneck events. The strategy we chose favors the emergence of highly fit mutants that can outcompete other potentially resistant mutants in the same culture dish. If one were interested in all theoretically possible resistance mechanisms without regard to competitive viability, performing selections in many small volume cultures would allow unfit parasites a larger chance of success due to decreased risk of competition. Other resistance selection strategies and their likely outcomes are reviewed in the literature (27, 28).

In the initial primary selections, wild-type parasites were selected with PfDHODH inhibitors. We used 3D7 or Dd2 parasites with identical sequences of a single copy of the *pfdhodh* gene. The resistant parasites that emerged from these selections were then tested against a panel of DHODH inhibitors for cross-resistance. Surprisingly, we identified several compounds that were more potent against the mutant, resistant parasites than their wild-type parents. We then performed two sets of selections as follows: sequential selections, where a drug-resistant mutant parasite was treated with a mutant-selective inhibitor, and simultaneous selections, where wild-type parasites were treated with a synergistic combination of wild-type and mutant-type selective inhibitors. The combination selection should target the most-fit resistance pathways and therefore block their emergence and spread. Thus, the combination will maintain a largely wild-type population that remains sensitive to wild-type PfDHODH inhibitors. We also identified compounds that were synergistic with wild-type PfDHODH inhibitors but did not exhibit mutually incompatible resistance. We predicted that these combinations would not protect from resistance.

##### Pathways to Drug Resistance

**FIGURE 1.**
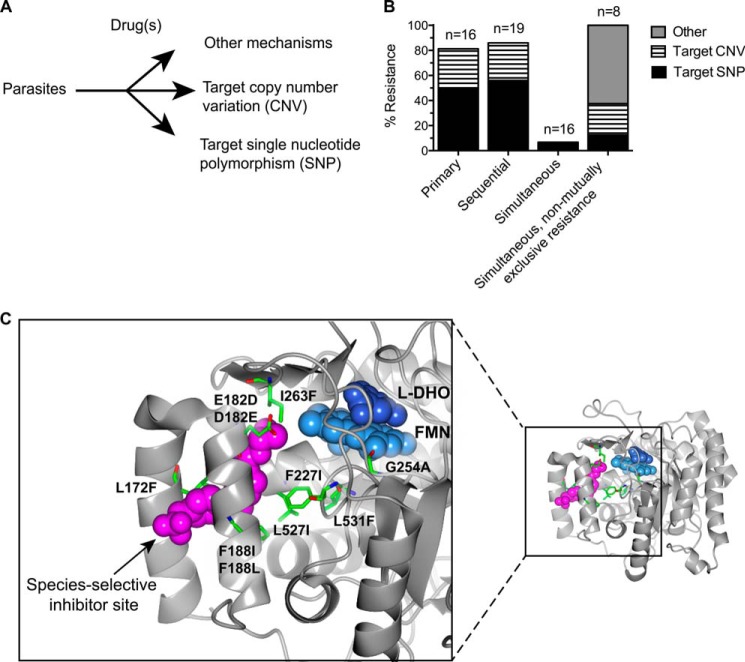
***In vitro* resistance selection results.** *A*, hypothetical resistance mechanisms include resistance by mutation or amplification of the target or a broad other category. *B*, observed resistance mechanisms were largely due to a SNP in the target. Target copy number changes and other mechanisms also led to resistance. Simultaneous selection with inhibitors with mutually incompatible resistance mechanisms greatly suppressed, but did not eliminate, the emergence of resistance. *C*, mutations in the target gene that confer resistance line the inhibitor-binding site. Image of Protein Data Bank 3o8a made in CCP4mg is shown (24). Residue 61 (for the N61S resistance mutation) is not part of the crystallography construct and so cannot be shown.

There are three theoretically possible mechanisms of resistance (Fig. 1, *A* and *B*) as follows: mutation in the target, copy number variation of the target, or a broad “other” category that could include adopting a quiescent state, increasing the quantity or activity of multidrug efflux pumps, altered drug metabolism, or altering pyrimidine biosynthetic metabolism or use through some other means.

In six sets of primary selections, we identified five instances of wild-type target gene amplification and six instances of single nucleotide polymorphisms (SNPs) in PfDHODH as follows: E182D (twice), F188I, F188L, F227I, and L531F (Table 3). Notably, the E182D mutation arose in response to selection with two structural classes of inhibitors, the triazolopyrimidines (DSM74) and the alkylthiophenes (Genz-666136). The related mutations F188I and F188L suggest that variation in codon 188 carries little fitness cost. Phe-227 and Leu-531 are highly conserved residues across DHODH enzymes from different species (23), so it is surprising that mutation in these positions results in viable parasites. In contrast, F188 is a variable residue. In humans, position 188 is a valine. This suggests that large and small hydrophobic residues are acceptable and that mutation to leucine or isoleucine would have little effect on enzyme activity and parasite viability.

**TABLE 3:** **Primary resistance selection results** NA means not applicable; ND means not determined.

Selection information				PfDHODH			PfMDR1 copy no.
Parasite	Drug(s)	Culture	Resistance	Residue	Nucleotide	Copy no.	
3D7	Genz-669178	1	Yes	WT	NA	2	1
		2	No	WT	NA	1	ND
		3	No	WT	NA	1	ND
		4	No	WT	NA	ND	ND
Dd2	Genz-669178	1	No	WT	NA	ND	ND
		2	Yes	F188I	T562A	1	4
		3	No	WT	NA	ND	ND
		4	Yes	F188L	T564A	1	2
3D7	Genz-666136	1	Yes	E182D	A516T	1	1
3D7	DSM74	1	Yes	E182D	A516T	1	1
Dd2	Genz-666136	1	Yes	F227I	T687A	1	2
Dd2	DSM74	1	Yes	L531F	G1593T	1	2
3D7 2× *pfdhodh* copies	Genz-669178	1	Yes	WT	NA	7	1
		2	Yes	WT	NA	8	2
		3	Yes	WT	NA	4	1
		4	Yes	WT	NA	4	1

Four sets of sequential selections gave target gene amplification twice and SNPs in PfDHODH nine times (Table 4). The L172F mutation rose to fixation in all four independent cultures of a single selection. Likewise, the L527I mutation dominated in four independent cultures of a different selection. Although we cannot assume that these mutations were not present in the starting population, all selections began with early freezes of cloned parasites to reduce this risk. One sequential selection gave rise to a wild-type revertant at the amino acid level (E182D/D182E), although the nucleotide sequence was different (GAA parent to GAT resistant mutant to GAG “revertant” double mutant).

**TABLE 4:** **Sequential resistance selection results** NA means not applicable; ND means not determined.

Selection information				PfDHODH			PfMDR1 copy no.
Parasite	Drug(s)	Culture	Resistance	Residue	Nucleotide	Copy no.	
3D7 E182D	IDI-6273	1	No	WT	NA	ND	ND
		2	Yes	D182E	T546G	1	1
		3	No	WT	NA	ND	ND
		4	No	WT	NA	ND	ND
Dd2 F227I	GSK3	1	Yes	L172F	A516T	1	2
		2	Yes	L172F	A516T	1	2
		3	Yes	L172F	A516T	1	2
		4	Yes	L172F	A516T	1	2
Dd2 F227I	IDI-6253	1	Yes	L527I	T1579A	1	5
		2	Yes	L527I	T1579A	1	5
		3	Yes	L527I	T1579A	1	5
		4	Yes	L527I	T1579A	1	5
Dd2 F227I	IDI-6273	1	No	F227I	NA	1	ND
		2	Yes	F227I	NA	2	2
		3	Yes	F227I	NA	2	2
		4	No	F227I	NA	1	ND

Four sets of simultaneous selections gave one instance of resistance, and this was associated with a SNP in PfDHODH, I263F (Table 5). Combining wild-type and mutant-type selective inhibitors greatly suppressed the emergence of resistance but did not eliminate it.

**TABLE 5:** **Simultaneous resistance selection results, mutually exclusive resistance** NA means not applicable; ND means not determined.

Selection information				PfDHODH			PfMDR1 copy no.
Parasite	Drug(s)	Culture	Resistance	Residue	Nucleotide	Copy no.	
Dd2	Genz-669178 + GSK3	1	Yes	I263F	A787T	1	6
		2	No	WT	NA	ND	ND
		3	No	WT	NA	ND	ND
		4	No	WT	NA	ND	ND
Dd2	Genz-669178 + IDI-6253	1	No	WT	NA	ND	ND
		2	No	WT	NA	ND	ND
		3	No	WT	NA	ND	ND
		4	No	WT	NA	ND	ND
Dd2	Genz-669178 + IDI-6273	1	No	WT	NA	ND	ND
		2	No	WT	NA	ND	ND
		3	No	WT	NA	ND	ND
		4	No	WT	NA	ND	ND
Dd2	Genz-669178 + DSM74	1	No	WT	NA	ND	5
		2	No	WT	NA	ND	3
		3	No	WT	NA	ND	6
		4	No	WT	NA	ND	6

All PfDHODH inhibitors, whether selective for wild-type or mutant-type, are highly synergistic with other PfDHODH inhibitors (data not shown). As a control, we identified compound pairs with similar levels of synergy as determined by a modified fixed-ratio isobologram (29) that did not exhibit mutually incompatible resistance (data not shown). Simultaneous selection with synergistic drug pairs that lack mutually incompatible resistance led to resistance in eight independent attempts (Table 6). One selection gave rise to a double mutant in PfDHODH: N61S and G254A. Two resistant lines had copy number variation of the *pfdhodh* gene. The other five lines were resistant through unknown mechanisms.

**TABLE 6:** **Simultaneous resistance selection results, nonmutually exclusive resistance** NA means not applicable; ND means not determined.

Selection information				PfDHODH			PfMDR1 copy no.
Parasite	Drug(s)	Culture	Resistance	Residue	Nucleotide	Copy no.	
Dd2	Genz-669178 + Genz-668419	1	Yes	WT	NA	1	4
		2	Yes	WT	NA	1	5
		3	Yes	WT	NA	4	3
		4	Yes	WT	NA	2	10
Dd2	Genz-669178 + atovaquone	1	Yes	WT	NA	1	3
		2	Yes	N61S, G254A	A122G, G761C	1	4
		3	Yes	WT	NA	1	5
		4	Yes	WT	NA	1	7

Primary selection gave resistance in 81.25% of attempts, sequential selection, 86%, and simultaneous selection, only 6.67%. Importantly, simultaneous selection with synergistic compounds that do not exhibit mutually incompatible resistance led to resistance in 100% of attempts (Fig. 1*B*).

The resistance-associated SNPs lined the “species-selective inhibitor site” in PfDHODH (Fig. 1*C*) (23). These mutations are positioned to influence binding of the helix-turn-helix lid as well as the binding of ubiquinone, which is proposed to occur in the same site. Notably, triazolopyrimidine inhibitors such as DSM74 appear to block electron flow from FMN to ubiquinone but not from FMN to inorganic oxidants like atmospheric oxygen or ferricyanide (23). This finding suggests that the inhibitor site's native function is as a binding site and electron tunnel for ubiquinone and FMN.

##### Drug Responses of Resistant Parasite Lines

**FIGURE 2.**
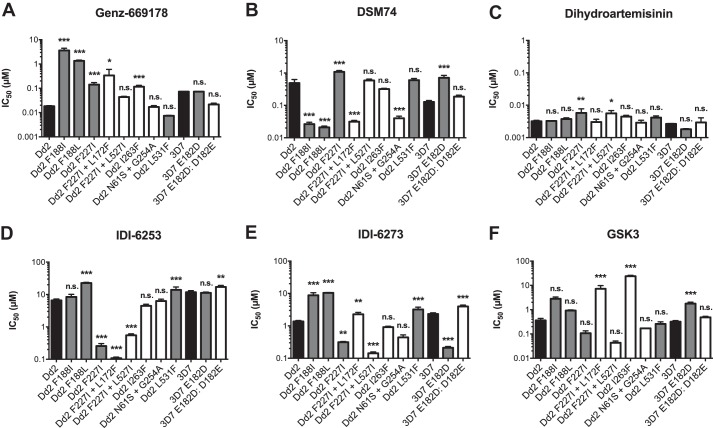
**Resistance to PfDHODH inhibitors via mutation in the target gene.** *A* and *B*, mutations in the target gene *pfdhodh* gave resistance to the wild-type-selective PfDHODH inhibitor Genz-669178 but gave both resistance and sensitivity to DSM74. *C*, sensitivity to the unrelated antimalarial dihydroartemisinin was largely unchanged. Although a small effect was seen with dihydroartemisinin in several parasite lines, we do not know if nucleotide flux or PfDHODH-related mitochondrial processes are involved in persistence or resistance to artemisinin-based drugs. *D–F*, mutations in PfDHODH gave resistance or sensitivity to mutant-selective PfDHODH inhibitors. *n.s.*, not significant (*p* > 0.5); *, *p* < 0.05; **, *p* < 0.01; ***, *p* < 0.0015.

The different pathways to resistance gave markedly different responses to drug pressure. SNPs in PfDHODH often led to large changes in drug sensitivity (Fig. 2 and Table 7). We observed 11 SNPs in PfDHODH as follows: N61S and G254A, E182D, E182D/D182E, F188I, F188L, F227I, F227I and L172F, F227I and L527I, I263F, and L531F. The F188L mutation increased the IC_50_ value for Genz-669178, a wild-type-selective PfDHODH inhibitor, from 18 to 1300 nm, a 72-fold increase. The similar F188I mutation led to a 200-fold increase. F227I and all derivatives were more sensitive to IDI-6253 (25.4-fold), and E182D and F227I were more sensitive to IDI-6273 (11- and 4.5-fold, respectively). I263F gave resistance to both selecting agents as follows: 6.7-fold resistance to Genz-669178 and 66.7-fold resistance to GSK3 (18 to 120 nm and 360 nm to 24 μm, respectively). The mutations F188I and F188L increased sensitivity to the wild-type-selective inhibitor DSM74 about 25-fold (21 and 26 nm compared with parental 490 nm, respectively). The addition of an L172F mutation to the F227I mutant line increased the sensitivity to DSM74 15.8-fold, from 490 to 31 nm. The L531F mutant gave only slight, nonstatistically significant resistance to the corresponding selection agent DSM74 (490 to 600 nm). Perhaps PfDHODH L531F has a survival advantage that does not directly relate to changes in drug IC_50_, such as increased catalytic efficiency.

**TABLE 7:** **Inhibition values for *P. falciparum* cell lines with mutations in *pfdhodh***

	IC_50_ of parasite proliferation (nm)*^a,b^*											
	Dd2	Dd2 F188I	Dd2 F188L	Dd2 F227I	Dd2 F227I L172F	Dd2 F227I L527I	Dd2 I263F	Dd2 N61S G254A	Dd2 L531F	3D7	3D7 E182D	3D7 E182D: D182E
*pfdhodh* copies	1	1	1	1	1	1	1	1	1	1	1	1
*pfmdr1* copies	3	4	2	2	2	5	6	4	2	1	1	1
Genz-669178	18 ± 1.1	3600 ± 830	1300 ± 110	140 ± 29	340 ± 260	44 ± 2.6	120 ± 16	17 ± 2.4	7.4 ± 0.32	13 ± 2.3	73 ± 0.63	22 ± 2.9
DSM74	490 ± 140	26 ± 3.7	21 ± 2.1	1080 ± 110	31 ± 3.0	590 ± 62	320 ± 16	40 ± 6.4	600 ± 76	130 ± 25	710 ± 130	190 ± 20
IDI-6253	6600 ± 690	8400 ± 1700	23000 ± 1100	260 ± 49	108 ± 8.9	550 ± 62	4500 ± 540	6300 ± 890	14000 ± 3200	12000 ± 1400	11000 ± 700	17000 ± 2000
IDI-6273	1400 ± 96	8800 ± 1700	10400 ± 310	310 ± 16	2300 ± 320	140 ± 14	920 ± 59	440 ± 86	3200 ± 580	2300 ± 202	210 ± 21	4000 ± 410
GSK3	360 ± 79	2800 ± 502	930 ± 55	108 ± 26	7200 ± 2600	43 ± 8.0	24000 ± 2300	170 ± 5.0	260 ± 56	320 ± 34	1800 ± 260	480 ± 47
Dihydro-artemisinin	3.2 ± 0.2	3.3 ± 0.039	3.7 ± 0.34	5.8 ± 2.0	3.02 ± 0.66	5.6 ± 1.2	4.5 ± 0.43	2.9 ± 0.53	4.2 ± 0.55	2.7 ± 0.079	1.8 ± 0.071	2.9 ± 1.1

*^a^* Errors represent the standard error of the fit for at least three determinations using 12 different drug concentrations.

*^b^* Antimalarial efficacy was calculated from dose-effect curves based on the SYBR Green method (18).

The unrelated antimalarial dihydroartemisinin was used as a control, although most resistant parasite lines showed no significant changes in sensitivity, F227I and F227I and L527I (but not F227I and L172F) both gave an ∼1.8-fold increase in IC_50_, and this reached significance. Copy number of the multidrug-resistant transporter gene *pfmdr1* did not correlate with dihydroartemisinin resistance, and there is no known link yet between pyrimidine pools or PfDHODH-influenced mitochondrial physiology and dihydroartemisinin resistance.

**FIGURE 3.**
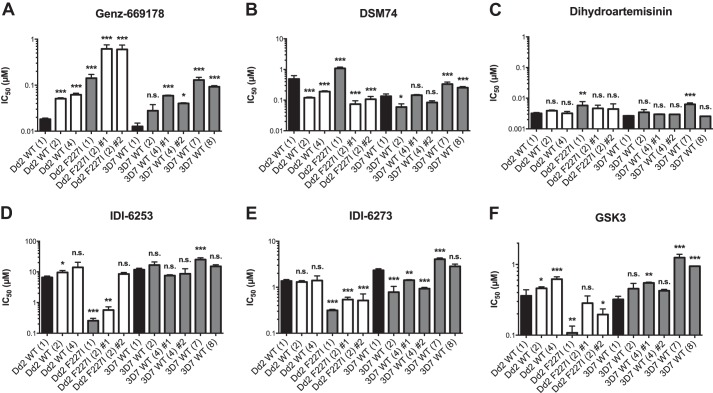
**Resistance to PfDHODH inhibitors through copy number variation of PfDHODH.** *A* and *B*, copy number variation of the target gene *pfdhodh* gave resistance to wild-type-selective PfDHODH inhibitors. Note that increased copies led to resistance for Genz-669178, an alkylthiophene, and to both resistance and sensitivity to DSM74, a triazolopyrimidine. *C*, copy number variation has little effect on the response to the unrelated antimalarial dihydroartemisinin. Although a small effect was seen with dihydroartemisinin in several parasite lines, we do not know if nucleotide flux or PfDHODH-related mitochondrial processes are involved in persistence or resistance to artemisinin-based drugs. *D–F*, copy number variation of PfDHODH gave resistance to the mutant-selective PfDHODH inhibitors IDI-6253 and GSK3, but occasionally gave sensitivity to IDI-6273. *n.s.*, not significant (*p* > 0.5); *, *p* < 0.05; **, *p* < 0.01; ***, *p* < 0.001.

Parasite lines with target gene amplification and no mutation in *pfdhodh* generally gave lower levels of resistance (Fig. 3 and Table 8). We observed parasites with 1, 2, 4, 7, or 8 copies of the *pfdhodh* gene. Two copies of *pfdhodh* in a 3D7 background increased resistance to the wild-type-selective inhibitor Genz-669178 2.15-fold, from 13 to 28 nm. The addition of a wild-type *pfdhodh* copy tended to give a linear increase in IC_50_, with 10–20 nm increases per copy. The addition of a mutant F227I copy gave a more-than linear increase, with IC_50_ values for Genz-669178 jumping from 140 nm (one copy of PfDHODH F227I) to 600–610 nm (two copies of PfDHODH F227I, two separate selections).

**TABLE 8:** **Inhibition values for *P. falciparum* cell lines with target copy number variation**

	IC_50_ of parasite proliferation (μm)*^a,b^*										
	Dd2	Dd2: Genz-669178 Genz-668419 no. 3	Dd2: Genz-669178 Genz-668419 no. 4	Dd2 F227I: IDI-6273 no. 2	Dd2 F227I: IDI-6273 no. 3	3D7	3D7: Genz-669178 no. 1	3D7 2× *pfdhodh:* Genz-669178 no. 1	3D7 2× *pfdhodh:* Genz-669178 no. 2	3D7 2× *pfdhodh:* Genz-669178 no. 3	3D7 2× *pfdhodh:* Genz-669178 no. 4
*pfdhodh* copies	1	4	2	2	2	1	2	7	8	4	4
*pfmdr1* copies	3	3	10	2	2	1	1	1	2	1	1
Genz-669178	18 ± 1.1	62 ± 5.0	51 ± 2.1	610 ± 140	600 ± 150	13 ± 2.3	28 ± 9.9	130 ± 19	93 ± 5.6	59 ± 1.7	40 ± 1.3
DSM74	490 ± 140	190 ± 11	120 ± 5.9	74 ± 21	110 ± 24	130 ± 25	59 ± 16	330 ± 55	260 ± 21	150 ± 6.8	84 ± 12
IDI-6253	6600 ± 690	14000 ± 6600	9600 ± 1500	630 ± 52	570 ± 160	12000 ± 1400	17000 ± 4300	25000 ± 3600	15000 ± 2000	7600 ± 490	8700 ± 4100
IDI-6273	1400 ± 96	1400 ± 360	1300 ± 86	540 ± 66	520 ± 200	2300 ± 202	780 ± 270	4100 ± 270	2800 ± 340	1400 ± 41	930 ± 59
GSK3	360 ± 79	620 ± 50	460 ± 24	280 ± 75	200 ± 39	320 ± 34	450 ± 86	1200 ± 150	950 ± 5.1	550 ± 13	420 ± 19
Dihydro-artemisinin	3.2 ± 0.21	3.2 ± 0.5	3.9 ± 0.21	4.6 ± 1.3	4.4 ± 2.1	2.7 ± 0.079	3.5 ± 0.78	6.3 ± 0.72	2.6 ± 0.03	3.0 ± 0.08	3.0 ± 0.07

*^a^* Errors represent the standard error of the fit for at least three determinations using 12 different drug concentrations.

*^b^* Antimalarial efficacy was calculated from dose-effect curves based on the SYBR Green method (18).

Parasites with multiple copies of PfDHODH: F227I were more sensitive to the wild-type-selective inhibitor DSM74 (IC_50_ of 490 nm dropping to 74 or 110 nm). F227I-amplified lines were also more sensitive to the mutant-selective inhibitors IDI-6253 (6600 to 630 or 570 nm) and IDI-6273 (1400 to 540 or 520 nm). Two copies of wild-type *pfdhodh* in a Dd2 background increased sensitivity to the wild-type-selective inhibitor DSM74, with IC_50_ values dropping from 130 to 59 nm, a 2.2-fold decrease. It is unclear how increasing gene dose increases sensitivity to certain compounds, although these parasite lines may certainly have unknown differences outside of *pfdhodh* copy number and sequence. Producing transgenic parasite lines in isogenic backgrounds could help dissect this point.

Increased sensitivity due to increased gene copy number suggests that buildup of PfDHODH products and/or the consumption of excessive amounts of starting materials are toxic to the cell. PfDHODH catalyzes a reversible set of half-reactions and is the rate-limiting step in pyrimidine biosynthesis, so a deleterious buildup of physiological products is unlikely. Excessive consumption of respiratory chain ubiquinone (oxidized to go forward and reduced to go in reverse), FMN, and l-dihydroorotate substrate may alter cell physiology, particularly in the single mitochondrion of the blood stage parasite. Finally, the inhibitors may act as substrates or react with the substrates in a way that poisons the cell. Careful analysis of *in vitro* enzymatic metabolites may be informative.

**FIGURE 4.**
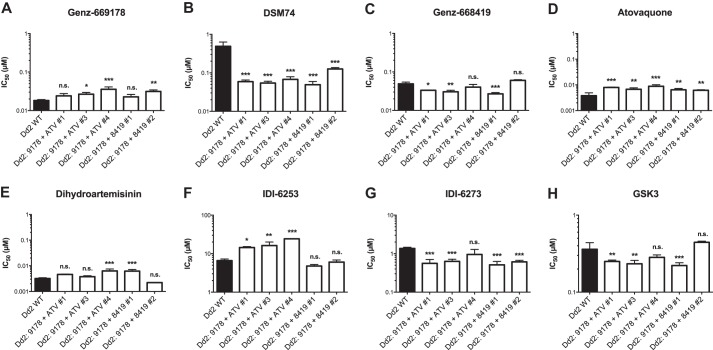
**Resistance to PfDHODH inhibitors without mutation or copy number variation in *pfdhodh*.** *A–C*, unknown resistance mechanisms altered responses to wild-type-selective PfDHODH inhibitors. *D*, several parasites, including two that were not selected with atovaquone, gained resistance to atovaquone. Atovaquone's target, cytochrome *bc*_1_, is important for PfDHODH function. *E*, sensitivity to the unrelated antimalarial dihydroartemisinin was largely unaffected. *F–H*, these parasites also had altered responses to mutant-selective PfDHODH inhibitors. *n.s.*, not significant (*p* > 0.5); *, *p* < 0.05; **, *p* < 0.01; ***, *p* < 0.001.

Finally, we identified five parasite lines that were resistant to PfDHODH inhibitors without mutation in or copy number variation of the *pfdhodh* gene (Fig. 4 and Table 9). All of these parasites were simultaneously selected with compounds without mutually exclusive resistance, and all had rather low fold changes in sensitivity. Of note, all five had an increased sensitivity to the wild-type-selective PfDHODH inhibitor DSM74, with IC_50_ values falling from 490 nm in the Dd2 parent to ∼55 nm in four lines and 130 nm in one line. The mechanisms of resistance are unknown. Two of these parasite lines showed slight resistance to dihydroartemisinin. Although these parasites had increased *pfmdr1* copies (four and five relative to the parental three copies), another parasite with seven copies of *pfmdr1* had no change in sensitivity. The parasites selected with atovaquone, a cytochrome *bc*_1_ inhibitor, had no mutations in cytochrome *b* or cytochrome *c* oxidase (data not shown). PfDHODH and cytochrome *bc*_1_ are both embedded in the inner mitochondrial membrane. PfDHODH requires functional cytochrome *bc*_1_ for generation and maintenance of a mitochondrial proton gradient, and thereby a supply of respiratory chain ubiquinone.

**TABLE 9:** **Inhibition values for *P. falciparum* cell lines with unknown mechanisms of resistance**

Compound	IC_50_ of parasite proliferation (μm)*^a,b^*						
	Dd2	Dd2: Genz-669178 + Genz-668419 no. 1	Dd2: Genz-669178 + Genz-668419 no. 2	Dd2: Genz-669178 + Atovaquone no. 1	Dd2: Genz-669178 + Atovaquone no. 2	Dd2: Genz-669178 + Atovaquone no. 3	Dd2: Genz-669178 + Atovaquone no. 4
*pfdhodh* copies	1	1	1	1	1	1	1
*pfmdr1* copies	3	4	5	3	4	5	7
Genz-669178	18 ± 1.1	36 ± 5.6	31 ± 3.1	24 ± 3.6	17 ± 2.4	23 ± 3.5	27 ± 2.9
Genz-668419	49 ± 5.5	40 ± 7.0	20 ± 2.8	33 ± 0.32	26 ± 1.5	27 ± 2.1	31 ± 2.9
Atovaquone	0.38 ± 0.11	8.9 ± 1.3	6.2 ± 0.29	7.9 ± 0.29	4.9 ± 0.34	6.4 ± 0.83	6.7 ± 0.96
DSM74	490 ± 140	67 ± 12	130 ± 11	59 ± 6.1	40 ± 6.4	49 ± 10.3	54 ± 6.1
IDI-6253	6600 ± 690	24000 ± 310	6000 ± 830	14,000 ± 830	6300 ± 890	4800 ± 480	16,000 ± 3800
IDI-6273	1400 ± 96	950 ± 340	610 ± 61	560 ± 150	440 ± 86	510 ± 120	630 ± 84
GSK3	360 ± 79	280 ± 21	440 ± 16	250 ± 11	170 ± 5.0	220 ± 19	230 ± 24
Dihydro-artemisinin	3.2 ± 0.21	6.3 ± 1.2	2.2 ± 0.05	4.6 ± 0.16	2.9 ± 0.53	6.2 ± 1.0	2.8 ± 0.32

*^a^* Errors represent the standard error of the fit for at least three determinations using 12 different drug concentrations.

*^b^* Antimalarial efficacy was calculated from dose-effect curves based on the SYBR Green method (18).

##### Evolutionary Loop, Reversion of Codon 182

The most striking finding of the selections was a reversion of a mutant parasite to wild type, which we have previously published (25). Selection of 3D7 wild-type parasites with either an alkylthiophene (Genz-666136) or triazolo-pyrimidine (DSM74) PfDHODH inhibitor led to the same point mutation in the target gene, E182D. This parasite was hypersensitive to the compound IDI-6273. Selection of the 3D7 E182D mutant line with IDI-6273 led to a resistant parasite with a second mutation in codon 182. This mutation encoded the alternate codon for the wild-type residue, glutamate. As the alternate glutamate codon (GAG) is used 9-fold less frequently than the major glutamate codon (GAA) in *P. falciparum*, it is possible that rare codon effects such as reduced mRNA stability and translation elongation pausing affect this “wild-type revertant” parasite (30).

Indeed, the 3D7 E182D/D182E revertant was not perfectly wild-type, although the sensitivity to some drugs was more similar to wild-type than the E182D mutant (Genz-669178, Genz-668419, GSK3), some were not. IDI-6273 changed from an IC_50_ of 2.3 μm in the 3D7 wild-type to 210 nm in the E182D mutant, an 11-fold increase in sensitivity. The wild-type revertant line had an IC_50_ of 4.0 μm for IDI-6273, 1.7-fold higher than 3D7 and 19-fold greater than the selection parent, 3D7 E182D. The “wild-type” revertant E182D/D182E is more similar to the 3D7 wild-type parasite than the 3D7 E182D selection parent (see Fig. 2 and Table 7). Whole genome sequencing of the 3D7 E182D/D182E revertant line (supplemental information) identified an I239V mutation in cytochrome *c* oxidase (PlasmoDB code mal_mito_1) relative to the 3D7 reference, but this mutation was also present in the 3D7: E182D line. Cytochrome *c* oxidase I239V may be important as PfDHODH activity relies on electron flow to and from the mitochondrial electron transport chain.

**FIGURE 5.**
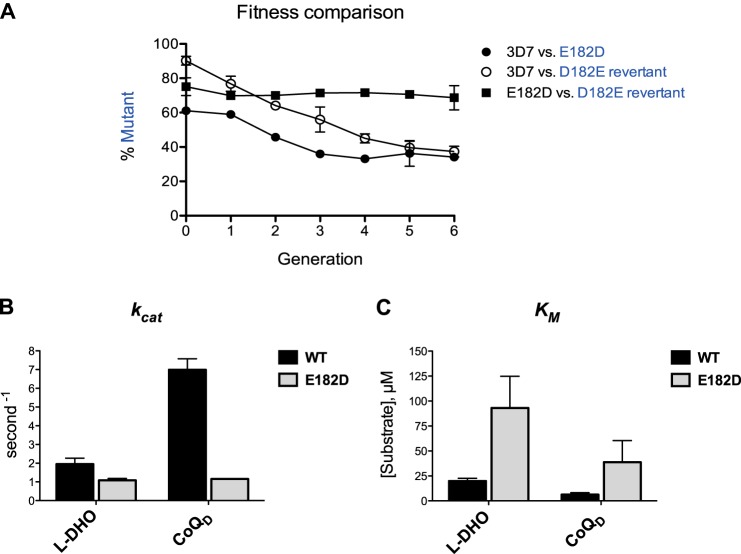
**Evolutionary loop in *pfdhodh* codon 182.** *A*, 3D7: E182D mutant and 3D7 E182D/D182E revertant were approximately equal in fitness to each other, but both were less fit than the wild-type 3D7. Note that the rate of decrease for the initial three cycles is greater than for the subsequent three cycles. *B* and *C*, purified PfDHODH protein with codon 182 mutated to aspartate showed substantially lower *k*_cat_ and *K_m_* values than the wild-type protein. This suggests that the reduced fitness of the 3D7 E182D mutant may be due to a less catalytically efficient enzyme, but it does not explain the fitness cost seen in the 3D7 E182D/D182E revertant. We propose that the revertant has reduced fitness due to rare codon effects and/or changes outside of the *pfdhodh* gene.

We measured comparative growth rates by growing all pairwise combinations of these three cell lines. Over six generations, both the E182D mutant and the E182D/D182E revertant declined to about 40% prevalence when grown with 3D7 and then held steady, indicating altered growth rates in both mutants relative to wild type (Fig. 5*A*). However, when the two mutants were grown together, no change was seen in relative allele frequency, indicating roughly equal fitness in the conditions tested. It is possible that the E182D mutation and the effects of a rare codon in the E182D/D182E revertant line counterbalance each other. Organismal fitness could be further assessed by determining cell cycle time and burst size by flow sorting. Protein stability could be assessed via ribosomal profiling and/or Western blot, although this task is hampered by the lack of commercially available PfDHODH antibodies.

We determined the steady-state parameters of recombinant wild-type and E182D PfDHODH protein by directly measuring the production of orotate product (Fig. 5, *B* and *C*). The E182D mutation decreased *k*_cat_ and increased *K_m_* values for both the l-dihydroorotate (l-DHO) substrate and the CoQ_d_ co-substrate, although it should be noted that the E182D mutant protein appears to degrade more easily than the wild-type protein and is thus not a homogeneous species. We do not know if differences observed between wild-type and E182D proteins are due to differences in enzyme activity and/or stability. Additionally, expression of full-length protein that may better reflect physiology is not yet possible due to an amino-terminal membrane anchor and mitochondrial targeting sequence that are deleted for all protein work shown. Enzyme kinetic changes were all less than 6-fold and are clearly compatible with viability in culture.

##### Drug Responses of Purified Protein Mirror Those of Resistant Cell Lines

**FIGURE 6.**
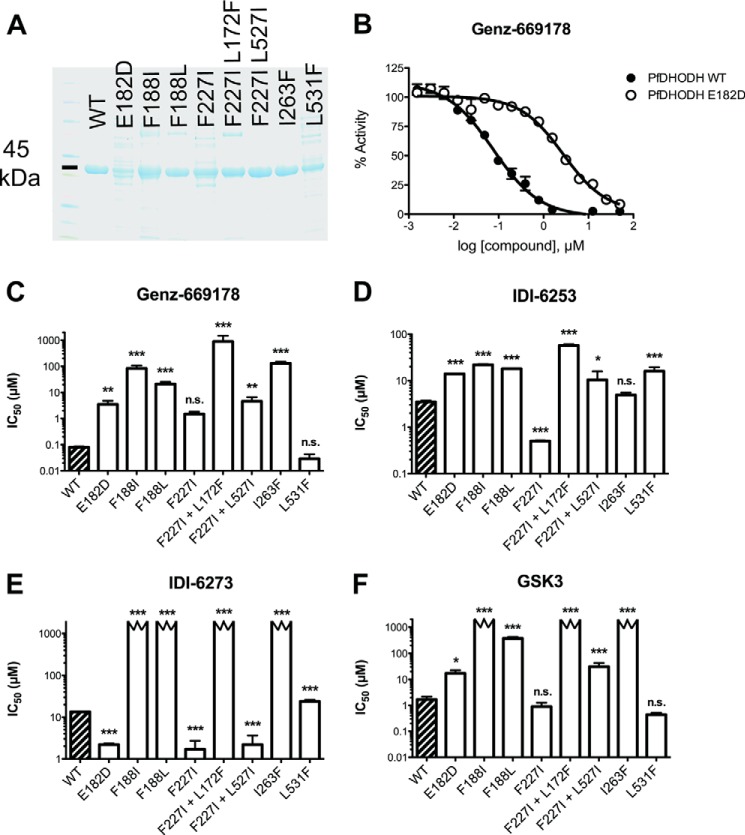
**Enzymatic inhibition mirrors whole cell activity of PfDHODH inhibitors.** *A*, Coomassie-stained gel indicates purity of the various purified PfDHODH proteins. *B*, sample data for enzymatic inhibition curves show a rightward shift (resistance) of the E182D protein to Genz-669178, which matches the whole cell data. *C–F*, enzymatic inhibition correlated with whole cell proliferation inhibition, which suggests but does not prove that mutation in PfDHODH causes the observed changes in sensitivity to PfDHODH inhibitors. *Cutoff bars* indicate an inhibition value greater than the maximum limit of detection of the assay, 2 mm.

We cloned and expressed codon-optimized PfDHODH proteins with engineered resistance mutations (Fig. 6 and Table 10). We used an indirect coupled indicator dye assay to determine the effect of PfDHODH inhibitors on protein activity. This assay reflected cellular results, suggesting that mutations in the *pfdhodh* gene are the driving factor in resistance and not mere coincidence. For example, inhibition of E182D protein activity required more Genz-669178 compound than wild-type protein, which is also true in cells (Fig. 6*B*). The fit between protein and cell data was substantially closer to the wild-type than many of the mutants, indicating that the *in vitro* protein activity assays may be missing components that affect mutant PfDHODH in cells, such as protein-folding chaperones or biological membranes to cross. Interestingly, many of the inhibitors were more potent in cellular assays than against purified protein. This indicates that these inhibitors may have multiple targets, active metabolites in cells, and/or a cellular accumulation effect. Several mutant proteins were not inhibited by the maximum amount of drug permissible in the assay (2 mm).

**TABLE 10:** **Inhibition values for wild-type and mutant-type purified enzyme**

Compound	IC_50_ of enzyme activity (μm)*^a,b^*								
	WT	F188I	F188L	E182D	F227I	F227I + L172F	F227I + L527I	I263F	L531F
Genz-669178	0.08 ± 0.006	83 ± 26	21 ± 4.5	3.5 ± 1.3	1.5 ± 0.33	890 ± 590	4.6 ± 2.002	130 ± 21	0.029 ± .014
DSM74	0.38 ± 0.09	1.03 ± 0.003	0.36 ± 0.028	19 ± 2.9	6.7 ± 2.7	30.4 ± 6.6	7.5 ± 3.09	85 ± 36	5.6 ± 0.32
IDI-6253	3.5 ± 0.22	22 ± 0.80	18 ± 0.29	14 ± 0.33	0.502 ± 0.019	57 ± 37	10.4 ± 5.5	4.9 ± 0.67	16 ± 3.4
IDI-6273	13.5 ± 0.32	>2000	>2000	2.2 ± 0.13	1.7 ± 1.02	>2000	2.2 ± 1.4	>2000	24 ± 2.0
GSK3	1.7 ± 0.46	>2000	370 ± 55	17 ± 5.4	0.89 ± 0.37	>2000	31 ± 12	>2000	0.44 ± 0.086

*^a^* Errors represent the standard error of the fit for at least three determinations using 12 different drug concentrations.

*^b^* IC_50_ values were determined using a continuous assay where the reagent concentrations were 200 μm l-DHO, 18 μm CoQ_d_, 100 μm dichloroindophenol, and 2–10 nM DHODH.

##### Protein Crystallography Shows Structural Flexibility in the Inhibitor-binding Site

**FIGURE 7.**
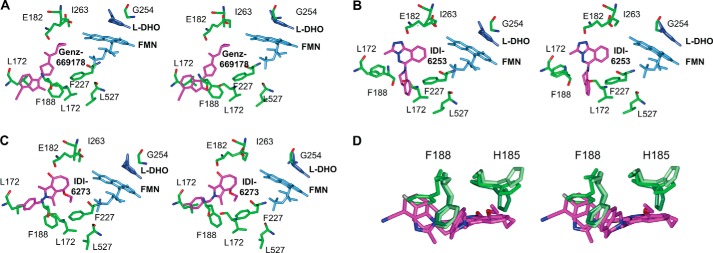
**Structural plasticity in the PfDHODH inhibitor-binding pocket.** *A–C*, stereo images of PfDHODH crystal structures with ligands Genz-669178 (*A*), IDI-6253 (*B*), and IDI-6273 (*C*) are shown (structures 4cq8, 4cq9, and 4cqa, respectively). *D*, two residues, histidine 185 and phenylalanine 188, have substantially altered conformations upon ligand binding. Note that Phe-188 was mutated twice in resistance selections for PfDHODH inhibitors (F188I and F188L). Images were made in CCP4mg (24).

In an attempt to better understand selectivity between and resistance to PfDHODH inhibitors, we performed co-crystallography experiments. We crystallized wild-type PfDHODH with the following three inhibitors: Genz-669178, IDI-6253, and IDI-6273 (Fig. 7 and Table 11). The inhibitor-binding site is highly plastic, as has been observed previously (22). Residues Phe-188 and His-185 moved substantially to accommodate the three different ligands (Fig. 7*D*), and subtle changes in helix packing slightly shifted other residues. His-185 is one of only two polar residues to contact the ligands (the other is Arg-265). Mutation of Phe-188 to leucine or isoleucine gave high level resistance to wild-type PfDHODH inhibitors, so it is interesting that Phe-188 is so flexible. Two main possibilities could explain this: one, the large aromatic ring of phenylalanine has more favorable interactions with the inhibitors than the small aliphatic chains of leucine and isoleucine, perhaps *pi* stacking or edge-to-face interactions. Two, Phe-188 acts as a gatekeeper to the inhibitor-binding site tunnel, and the small residues do not hold the inhibitors in place.

**TABLE 11:** **X-ray crystallography parameters**

Compound	ID	Resolution	*R*	*R*-free	Space group	No. of molecules	Unit cell					
		Å										
Genz-669178	4cq8	2.0	0.170	0.190	C2	2	147.820	69.130	103.850	90.000	103.840	90.000
IDI-6253	4cq9	2.8	0.171	0.230	P2_1_	2	52.950	166.410	63.160	90.000	107.910	90.000
IDI-6273	4cq8	2.7	0.194	0.268	P2_1_	2	53.762	135.445	67.873	90.000	89.640	90.000

I263F and the combination G254A and N61S both arose after simultaneous selection with two compounds. I263F came from the combination of Genz-669178, a wild-type-selective inhibitor, and GSK3, a mutant-selective inhibitor. From the Genz-669178 structure (see *ribbon diagram* in Fig. 1*C*), we can speculate that the bulky phenylalanine substitution in I263F may prevent multiple structural classes of PfDHODH inhibitors from binding, whether through direct steric clashes, altered helix packing, or an altered path of electron flow.

The G254A/N61S double mutant was generated through simultaneous selection with Genz-669178, a wild-type-selective PfDHODH inhibitor, and atovaquone, which targets the cytochrome *bc*_1_ complex. PfDHODH N61 is not part of the crystallography construct, but it is predicted to lie inside the mitochondrial matrix and thus may have a role in signaling, recruiting ubiquinone or other substrates, and so on. Gly-254 is present on a loop that is close to the active site. Although glycine to alanine is a conservative change, the addition of a methyl group may alter loop flexibility.

In general, mutations near the top or bottom of the amino-terminal helix-turn-helix lid (F188I, F188L, and I263F, see Fig. 1*C*) tended to give high level resistance with little apparent consequence. Mutations lining the presumed electron tunnel (E182D, F227I, L531F, and L527I) tended to give low level resistance, increased susceptibility to other compounds, and an anecdotal reduced protein stability. Tunnel mutations appear more liable to compromise enzyme function than lid mutations, and thus have graver consequences.

## DISCUSSION

Drug resistance can rapidly compromise the effective useful lifetime of antimalarial agents, and cross-resistance with existing therapies is a major concern as few compounds with novel mechanisms of action are being developed. However, drug resistance emerges in the context of a population and is limited by evolutionary fitness costs. If a parasite is drug-resistant, but has a high fitness cost in the absence of drug, that parasite is unlikely to be competitively viable. The vast majority of a *Plasmodium* parasite life cycle is spent in the absence of drug, whether because an infected person is asymptomatic and does not seek treatment or the infected person is ill but has no access to effective antimalarial therapies.

Fitness limitations result in a very small number of mutational escape pathways being heavily favored in *Plasmodium* (5) and likely in many other organisms as well. These escape pathways can be anticipated and blocked.

PfDHODH is a promising antimalarial drug target. In this study, we sought to characterize mechanisms of resistance to PfDHODH inhibitors and develop strategies to suppress the emergence and spread of resistance. Sixteen sets of resistance selections gave 11 point mutations in PfDHODH as follows: N61S, L172F, E182D, E182D/D182E, F188I, F188L, F227I, G254A, I263F, L527I, and L531F. Several of these mutations arose multiple times in independent selections. Ribosome profiling and the development of specific antibodies would be useful tools to assess the stability of these mutant transcripts and proteins. Protein stability and flexibility could also be assessed through solution NMR-based structural studies. Gene amplification and unknown mechanisms also contributed to resistance, but these were less common and resulted in lower levels of resistance. Obtaining two copies of the *pfdhodh* gene was rare, but further selection of parasites that had two copies led to rapid expansions to four, seven, or eight copies.

Although *in vitro* culture lacks important elements of human infection such as an immune response, *in vitro* selections have identified several resistance pathways that were also observed in human patients (7, 31), *e.g.* multidrug-resistance transporter 1 (PfMDR1) as the main resistance locus for mefloquine (32) and mutations in the cytochrome *bc*_1_ complex as the main resistance locus for atovaquone (33).

The activity of purified protein mirrored cellular data, which support the idea that alterations in *pfdhodh* cause resistance to PfDHODH inhibitors. Allelic exchange in isogenic parasites could confirm this, but *pfdhodh* has been refractory to traditional genetic approaches.

Crystallography showed substantial plasticity in the inhibitor site but also suggested that selectivity between wild-type and mutant-type selective inhibitors is largely driven by small changes in hydrophobic interactions due to altered helix packing. Wild-type-selective inhibitors tend to bind much closer to the FMN cofactor than mutant-type-selective inhibitors. The observation of cross-resistance to some PfDHODH inhibitors and hypersensitivity to others in resistant parasites implies that the inhibitor site has a limited mutational flexibility, likely limited by fitness constraints.

The identification of mutant-selective inhibitors allows us to set a trap; if resistance to one compound results in hypersensitivity to another, then a parasite will be more fit if it retains wild-type sensitivity. We call this concept “targeting resistance,” as the resistance allele itself becomes a drug target. Targeting resistance relies on stepwise acquisition of resistance. If the parasite is able to become resistant to both compounds in a single step, such as the PfDHODH I263F mutation, targeting resistance would be much more likely to fail. The PfDHODH inhibitors shown all bind in overlapping but distinct sites. Although the incidence of resistance was much lower in combination selections than in single-compound selections, it was not zero. An improved combination set would target binding sites that do not influence each other. A distant site on the same target or another target entirely would be ideal. Different targets would be far less susceptible to resistance through copy number variation or single mutations. Conversely, overlapping binding sites allows the possibility of a hybrid double-warhead molecule that is active against both wild-type and mutant-type parasites. A bifunctional molecule would ease many practical pharmacology and toxicology concerns.

The concept of targeting resistance has already been validated by a long term natural experiment, chloroquine and lumefantrine. Widespread use of chloroquine led to resistance. Chloroquine resistance led to the use of other antimalarial drugs, such as lumefantrine-artemether (Coartem). Surprisingly, chloroquine-resistant parasites were hypersensitive to lumefantrine. This induced sensitivity was traced to mutually incompatible resistance mechanisms (32); *pfcrt* and *pfmdr1* alleles and copy numbers that gave resistance to chloroquine resulted in sensitivity to some other classes of drugs, including lumefantrine (34, 35). As a corollary, the fitness costs that many chloroquine resistance alleles carry and the widespread use of lumefantrine as an artemether partner drug have resulted in the return of chloroquine-sensitive parasites in several locales (36).

An area for improvement in the targeting resistance strategy would be to identify mutant-specific (rather than mutant-selective) compounds. The mutant-selective compounds have relatively little activity against the wild type, but it is not negligible. This selective force can lead to resistance developing in the wild-type population, which could compromise the effectiveness of the mutant-selective compound when needed. The wild-type population is large and thus has a large potential for diversity. The emerging resistant population is small and thus has relatively little diversity. The less time the nascent resistant population is allowed to exist, the less time it has to accumulate diversity and thus develop competitively viable resistance. Therefore, the goal is to neutralize emerging threats as soon as possible, before they have a chance to spread or become more fit through compensatory mutations. Identifying mutant-specific inhibitors would eliminate selective pressure for resistance in the wild-type population. Another strategy is to identify inhibitors that are highly active against both wild-type and mutant-type parasites, as was neatly demonstrated for *P. falciparum* dihydrofolate reductase (37).

By targeting both the wild-type population and the most fit resistance pathways, we can accomplish two goals, kill parasites and help prevent the emergence of resistance. Selecting partner drugs or creating double-warhead molecules that block resistance increases the useful lifetime of an antimalarial agent, and it should be considered before putting therapeutics in clinical use.

## Supplementary Material

Supplemental Data

## Footnotes

2 The abbreviations used are:

DHODH: dihydroorotate dehydrogenase
MT: mutant-type
l-DHO: l-dihydroorotate
CoQ_d_: decylubiquinone
SNP: single-nucleotide polymorphism
FMN: flavin mononucleotide
PfMDR1: *P. falciparum* multidrug resistance transporter 1.
