# Supplementary material for: In Vitro Resistance Selections for Plasmodium falciparum Dihydroorotate Dehydrogenase Inhibitors Give Mutants with Multiple Point Mutations in the Drug-binding Site and Altered Growth
Source: J Biol Chem. 2014 Apr 29;289(26):17980–95. doi: 10.1074/jbc.M114.558353 (PMC4140291; doi:10.1074/jbc.M114.558353)
Supplement: Supplemental Data [file supp_289_26_17980__index.html]

In Vitro Resistance Selections for Plasmodium falciparum Dihydroorotate Dehydrogenase Inhibitors give Mutants with Multiple Point Mutations in the Drug-Binding Site and Altered Growth — In Vitro Resistance Selections for Plasmodium falciparum Dihydroorotate Dehydrogenase Inhibitors Give Mutants with Multiple Point Mutations in the Drug-binding Site and Altered Growth — Resistance to DHODH Inhibitors in Malaria — Supplemental Data 

# *In Vitro* Resistance Selections for *Plasmodium falciparum* Dihydroorotate Dehydrogenase Inhibitors Give Mutants with Multiple Point Mutations in the Drug-binding Site and Altered Growth

## Supplemental Data

**Files in this Data Supplement:**

- Whole genome sequencing results (.xlsx, 2.8 MB)
